# Clusters of apoptotic signaling molecule-enriched rafts, CASMERs: membrane platforms for protein assembly in Fas/CD95 signaling and targets in cancer therapy

**DOI:** 10.1042/BST20211115

**Published:** 2022-05-19

**Authors:** Faustino Mollinedo, Consuelo Gajate

**Affiliations:** Laboratory of Cell Death and Cancer Therapy, Department of Molecular Biomedicine, Centro de Investigaciones Biológicas Margarita Salas, Consejo Superior de Investigaciones Científicas (CSIC), Ramiro de Maeztu 9, E-28040 Madrid, Spain

**Keywords:** apoptosis, cancer therapy, Fas/CD95, lipid rafts, signalling

## Abstract

Mammalian cells show the ability to commit suicide through the activation of death receptors at the cell surface. Death receptors, among which Fas/CD95 is one of their most representative members, lack enzymatic activity, and depend on protein–protein interactions to signal apoptosis. Fas/CD95 death receptor-mediated apoptosis requires the formation of the so-called death-inducing signaling complex (DISC), bringing together Fas/CD95, Fas-associated death domain-containing protein and procaspase-8. In the last two decades, cholesterol-rich lipid raft platforms have emerged as scaffolds where Fas/CD95 can be recruited and clustered. The co-clustering of Fas/CD95 and rafts facilitates DISC formation, bringing procaspase-8 molecules to be bunched together in a limited membrane region, and leading to their autoproteolytic activation by oligomerization. Lipid raft platforms serve as a specific region for the clustering of Fas/CD95 and DISC, as well as for the recruitment of additional downstream signaling molecules, thus forming the so-called cluster of apoptotic signaling molecule-enriched rafts, or CASMER. These raft/CASMER structures float in the membrane like icebergs, in which the larger portion lies inside the cell and communicates with other subcellular structures to facilitate apoptotic signal transmission. This allows an efficient spatiotemporal compartmentalization of apoptosis signaling machinery during the triggering of cell death. This concept of proapoptotic raft platforms as a basic chemical-biological structure in the regulation of cell death has wide-ranging implications in human biology and disease, as well as in cancer therapy. Here, we discuss how these raft-centered proapoptotic hubs operate as a major linchpin for apoptosis signaling and as a promising target in cancer therapy.

## Introduction

A remarkable feature of mammalian cells lies in the presence of death receptors at the cell surface, ready to induce their own cell death by apoptosis upon appropriate activation. Physiologically, this ability to commit suicide is mediated by the interaction of a death receptor, a member of the tumor necrosis factor (TNF)-receptor superfamily (TNFRSF), with its cognate ligand. This extrinsic apoptotic pathway depends on the external stimulation following the binding of extracellular death ligands, such as Fas ligand (FasL, CD95L, CD178, TNSF6), tumor necrosis factor-α (TNF-α, cachectin, TNFSF2) and/or TNF-related apoptosis-inducing ligand (TRAIL, CD253, APO-2L, TNFSF10), to their cognate death receptors Fas (CD95, APO-1, TNFRSF6), TNF-receptor 1 (TNF-R1, CD120a, TNFRSF1A), TRAIL-receptor 1 (TRAIL-R1, CD261, DR4, APO-2, TNFRSF10A), and/or TRAIL-R2 (CD262, DR5, TNFRSF10B), respectively, on the plasma membrane [1–3], which are promising targets in cancer [4]. Fas/CD95 is one of the key activators of the extrinsic apoptotic pathway.

Human mature Fas/CD95 is a 45–48 kDa (319 amino acids) type I transmembrane receptor, with a 157-amino acid N-terminal extracellular region containing three cysteine-rich domains, a single transmembrane domain of 17 amino acids, and a 145-amino acid C-terminal cytoplasmic region. The cytoplasmic portion of Fas/CD95 has no enzymatic activity, but contains a ∼88-amino acid domain termed the death domain (DD), which is critical for transmitting the apoptotic signal and is homologous to similar domains in other death receptors [5–8]. Because death receptors lack enzymatic activity, their function in signal transmission depends on protein–protein interactions through specific domains, such as the DD, which has a strong propensity to self-associate [6–9].

Fas/CD95 is homotrimerized before activation, and the interaction with FasL/CD95L leads to a next level of its aggregation, leading to signal transmission [8]. The binding of FasL/CD95L promotes a series of critical sequential events, namely: (a) potentiates and stabilizes the trimeric receptor conformation, through a proline-containing motif in the Fas/CD95 transmembrane region that favors Fas/CD95 tight packing by close van der Waals interactions between a small proline side chain and an isoleucine amino acid in the adjacent monomer [10]; (b) prompts aggregation and oligomerization of adjacent Fas/CD95 trimers, which can be largely affected by the lipid composition of the plasma membrane as well as by certain lipids [11–13]; (c) induces the recruitment of the adaptor protein Fas-associated death domain-containing protein (FADD), through homotypic interactions between the DDs of both Fas/CD95 and FADD, which is crucial for the subsequent recruitment of procaspase-8 and the ensuing cascade of events leading to apoptosis [6]. This latter interaction between FADD and procaspase-8 is also mediated by homotypic interactions between both proteins through their respective death effector domains (DEDs) [14]. As a result of the above homotypic interactions through DD and DED domains, activation of Fas/CD95 leads to the generation of the so-called ‘death-inducing signaling complex’ (DISC) [15,16], made up of Fas/CD95, FADD and procaspase-8, which is the critical initial step of Fas/CD95 apoptotic signaling [17]. Activation of the procaspase-8 at the DISC turns procaspase-8 into mature active caspase-8 by autoproteolysis, which eventually triggers the downstream and execution steps of apoptosis. Quantitative mass spectrometry, Western blot analysis and mathematical modeling show that procaspase-8 molecules outnumber (up to 9-fold) those of FADD in the DISC [18,19]. Procaspase-8 contains two DED motifs in tandem, so that each molecule can interact with FADD and other procaspase-8 molecules at the same time, or with two procaspase-8 molecules through the two tandemly arranged DED motifs. In this regard, procaspase-8 activation at the DISC is driven by DED filament assembly, containing several molecules of procaspase-8 in an appropriate molecular architecture to ensure two caspase-8 catalytic domains are correctly oriented to support activation, as recently shown by elegant cryo-electron microscopy structural analyses [20,21]. Procaspase-8 has a low basal enzymatic activity, but the high local concentration of oligomerized procaspase-8 in a limited area, brings procaspase-8 molecules in close proximity to each other, thus driving proximity-induced dimerization and proteolytic cleavage [22].

On the other hand, Fas/CD95 has been recently reported to interact with Kip1 ubiquitination-promoting complex protein 2 (KPC2) in triple-negative breast cancer cells, independently of FasL/CD95L, and leads to the partial degradation of NF-κB (nuclear factor kappa-light-chain-enhancer of activated B cells), thus suppressing NF-κB-driven gene expression [23], and pointing out the interplay of Fas/CD95 death receptor with an important cell survival signaling. This is of major importance for the well-known involvement of Fas/CD95 in both cell death and survival/inflammatory processes [24–26].

The presence of a membrane region where different proteins can gather would facilitate the above protein–protein interactions. With the dawn of the twenty-first century, the membrane domains known as lipid rafts were reported as putative scaffolds where the distinct processes leading to the triggering of death receptor-mediated apoptotic signaling could take place [27].

## Lipid rafts as mobile and sorting compartments in the membrane phospholipid bilayer

The initial view in 1972 of the plasma membrane as a double layer of lipids studded with proteins that were freely floating in the lipid sea (fluid mosaic model) [28], was refined in 1997 by the lipid raft hypothesis [29], following a series of studies that showed the presence of heterogeneous lipid domains and lipid sorting [30,31]. Lipid rafts behave as small (10–200 nm), heterogeneous and dynamic membrane regions, enriched in sphingolipids and cholesterol, resulting in liquid-ordered phases that display less fluidity than the surrounding plasma membrane [32–35]. The estimated average size for lipid rafts is ∼ 500 nm^2^, and larger platforms can be formed by clustering. A major advance in characterizing these lipid raft domains was their isolation as nonioinic detergent-resistant membranes (DRMs) at cold temperature, due to the higher proportion and tight packing of sphingolipids, cholesterol and saturated acyl chains of sphingolipids and phospholipids, as compared with the surrounding membrane [36]. The isolation of DRMs by flotation as low-density membranes in density gradients, such as sucrose gradients, is a major method to identify raft-associated proteins [6,37,38]. The use of different detergents has rendered DRMs with sundry protein and lipid compositions [39,40], supporting lipid raft heterogeneity [41,42]. Rafts have been found not only in the plasma membrane, but in extracellular vesicles and intracellular membranes, particularly involved in lipid and protein trafficking between the endoplasmic reticulum, Golgi and endosomes to the plasma membrane, and between the endoplasmic reticulum and mitochondria [43]. Cholesterol serves as a spacer between the hydrocarbon chains of the sphingolipids, having higher affinity to raft sphingolipids than to unsaturated phospholipids, and functions as a glue that keeps the raft assembly together [44,45]. The critical role of cholesterol in lipid rafts is clearly shown by the fact that removal of cholesterol renders them nonfunctional and leads to dissociation of proteins from rafts [9,45]. Diffusion of raft-associated proteins is significantly reduced compared with that of nonraft transmembrane proteins, a feature that is lost following cholesterol depletion. A hallmark of lipid rafts is their capacity to sort out proteins leading to the compartmentalization of different cellular processes and signaling pathways [9,44] through mechanisms not yet well understood. Thus, lipid rafts can be viewed as lipid scaffolds in which protein–protein interactions, required for a particular signaling process, can be favored [9]. In this regard, lipid rafts are becoming promising therapeutic targets [1,46–51]. Different approaches [6,52–55] have contributed in the last two decades to unravel the biophysical, kinetic and mechanical properties of these membrane domains, including the use of: lipid raft model membranes (e.g. ternary lipid mixtures containing cholesterol, sphingomyelin and phosphatidylcholine derivatives in different molar ratios, to form liquid ordered phases, which may coexist with other rigid or fluid phases, depending on the temperature); giant unilamellar vesicles; giant plasma membrane vesicles and plasma membrane spheres; novel fluorescence lipid analogs; single-molecule imaging; and additional high temporal and spatial resolution techniques. By using laser-based optical tweezers [56], liquid ordered domains, mimicking lipid rafts, were found to be moved, scattered, assembled, mixed and demixed [57].

## Cholesterol-rich lipid rafts in the triggering of Fas/CD95 apoptotic function

Lipid rafts were soon involved in a wide number of signal transduction processes related to the immune system and cell survival/proliferation [9,37,44], but their involvement in cell death processes was later found with the advent of this millennium. While investigating the mechanism of action of the antitumor ether lipid edelfosine (1-*O*-octadecyl-2-*O*-methyl-*rac*-glycero-3-phosphocholine, ET-18-OCH_3_), the prototype of a heterogeneous family of compounds collectively known as synthetic alkylphospholipid analogs [58–61], we found in 2001 that the induction of apoptosis triggered by this drug in leukemic cells was dependent on the translocation and co-clustering of Fas/CD95 into membrane rafts [27]. This led to the first demonstration of the recruitment of Fas/CD95 in lipid rafts, and the involvement of membrane rafts in Fas/CD95-mediated apoptosis and cancer chemotherapy [1]. The antitumor ether lipid edelfosine activated Fas/CD95 from the cytoplasmic side of the cell membrane, independently of FasL/CD95L [11,62]. This edelfosine-induced Fas/CD95-mediated apoptosis entailed the recruitment and clustering of Fas/CD95, FADD, and procaspase-8, thus forming the DISC, in clustered rafts [62–64]. Partial deletion of the Fas intracellular C-terminal domain, including part of the Fas/CD95 DD, prevented ether lipid-induced apoptosis [62,64]. Disruption of membrane rafts by cholesterol depletion with methyl-β-cyclodextrin (MβCD) [65], or cholesterol sequestration with the polyene macrolide antibiotic filipin [66], blocked edelfosine-induced Fas/CD95 aggregation and apoptosis [27,64]. In multiple myeloma cells, ceramide addition displaced cholesterol from rafts and inhibited the apoptotic response induced by the antitumor ether lipid [46]. Edelfosine-induced apoptosis in multiple myeloma cells was inhibited following raft disruption by cholesterol depletion *in vitro* [46,64], or by a decrease in tumor cell cholesterol *in vivo* [46]. However, cholesterol replenishment restored cell ability to undergo drug-induced apoptosis [46]. Several reports indicate that edelfosine accumulates in lipid rafts [1,46,62–64,67–69], and induces significant changes in lipid raft organization, altering their protein composition [1,46,47,62–64,68,70]. Edelfosine interacts with cholesterol in raft membrane models [71,72], increases membrane thickness, as seen by X-ray diffraction in model membranes containing 1-palmitoyl-2-oleoyl-*sn*-glycero-3-phosphocholine/sphingomyelin/cholesterol [71], and prompts a mild increase in membrane fluidity in model membranes [73]. These increases in membrane thickness and fluidity could facilitate the accommodation of a greater number of proteins as well as the in and out transport of proteins in membrane rafts. Interestingly, some cancer cells show elevated levels of lipid rafts and cholesterol [9,74,75]. On the other hand, the sialylated glycosphingolipids gangliosides are enriched in lipid rafts [76,77], and several tumor cells show characteristic ganglioside patterns [78,79]. The antitumor drug edelfosine has a strong affinity for ganglioside GM1 in model membranes [80]. The above evidence suggests that edelfosine behaves as a fascinating tool to identify cholesterol-enriched lipid rafts and to promote raft-mediated apoptotic processes in cancer cells. Soon after the identification of edelfosine-induced translocation and recruitment of Fas/CD95 into lipid rafts in a ligand independent way [27], indicating an essential role of membrane rafts in the initiation of Fas/CD95-mediated apoptosis [5,11,62], FasL/CD95L was also shown to promote the recruitment of the death receptor into rafts in mouse thymocytes, and cholesterol depletion abolished DISC formation and cell death [81]. Thus, it turned out that this recruitment of Fas/CD95 in lipid rafts and subsequent death receptor activation was a physiological process that could be triggered pharmacologically, and therefore it became a promising and appealing therapeutic target for cancer treatment [1]. Fas/CD95 can undergo a number of post-translational modifications, including its S-palmitoylation that facilitates its distribution in lipid rafts [6,8,82].

## Co-clustering of membrane rafts and Fas/CD95 as a target in cancer therapy

Time-resolved fluorescence resonance energy transfer (TR-FRET) studies on the ternary lipid system palmitoylsphingomyelin/1-palmitoyl-2-oleoyl-*sn*-glycero-3-phosphocholine/cholesterol, as a model for lipid rafts, showed that lipid rafts of very different sizes could be generated, depending on membrane composition and lipid–lipid interactions [83]. Studies in mammalian cells indicate that plasma membrane can contain lipid rafts showing different lipid packing and sizes [42,84]. Membrane rafts distribute in a very wide range, from the nanometer to the micrometer scale, with many factors influencing nucleation, domain growth, merging, diffusion, spinodal decomposition and spatial organization of raft components [85]. Thus, lipid rafts can be envisioned as mobile ice floes floating in a liquid-disordered lipid phase sea. However, they can associate to form larger raft platforms when proteins that associate with liquid-ordered domains oligomerize, or following changes in lipid composition.

Membrane rafts, as assessed by confocal microscopy using the raft marker fluorescein isothiocyanate-conjugated cholera toxin B subunit [86,87], which binds ganglioside GM1 [88], mainly found in rafts [89], were clustered in dense patches in hematological cancer cells upon treatment with the alkylphospholipid analog edelfosine [27,46,47,62]. These patches of membrane rafts accumulated Fas/CD95 clusters in leukemic cells treated with edelfosine, leading to a co-capping of rafts and Fas/CD95 as determined by confocal microscopy [27,46,47,62,64], which was further supported by the presence of Fas/CD95 in isolated rafts [27,62–64]. Edelfosine-induced co-clustering of Fas/CD95 and rafts, and subsequent induction of Fas/CD95-mediated apoptosis, were independent of the action of the FasL/CD95L [11,62]. The translocation of Fas/CD95 into lipid rafts was accompanied by the recruitment of FADD, procaspase-8, and procaspase-10 into rafts, forming the DISC and promoting apoptosis, as evidenced by functional, co-immunoprecipitation, genetic and fluorescence microscopy assays [62–64]. This evidence established lipid rafts as a novel therapeutic target and paved the way for a new avenue in the treatment of cancer, highlighting the role of lipid rafts in Fas/CD95-mediated apoptosis and cancer chemotherapy [1,9,27,62]. Despite edelfosine promoted the recruitment of procaspase-8 and procaspase-10 to rafts, the role of procaspase-10 in the initiation of Fas/CD95-mediated apoptosis is a matter of debate in contrast with procaspase-8. Caspase-10, which is absent from rodents [90], is a close homolog of caspase-8 and was found to be recruited to and processed in the DISC [91,92]. Both caspases have been considered to be redundant in their functions [93], sharing overlapping substrate specificities [94], but there is some controversy on whether caspase-10 can functionally substitute for caspase-8 in death receptor-mediated cell death [91,92]. Recent evidence indicates that caspase-10 negatively regulates caspase-8-mediated cell death, switching the FasL/CD95L-mediated response at the DISC level from caspase-8-induced cell death to NF-κB activation and cell survival [95].

An additional number of antitumor agents have been reported to promote apoptosis, at least in part, through the recruitment of death receptors in lipid rafts, including: cisplatin [96], oxaliplatin plus ursodexoycholic acid [97], rituximab [98], resveratrol [99,100], the alkylphospholipid analog perifosine [64], and the potent apoptotic inducer on leukemic cells aplidin/plitidepsin [101,102]. An updated list of agents that promote the recruitment of death receptors into lipid rafts is shown in [9]. However, edelfosine-induced Fas/CD95-raft remains as the epitome of this ligand-independent death receptor activation through the recruitment of death receptors in lipid rafts. Furthermore, it is worth noting the modulation of Fas/CD95-mediated apoptosis by lipid rafts in T lymphocytes [103,104], which may have important connotations for the pathogenesis and treatment of several diseases, including autoimmune diseases.

Shortly after the finding of the translocation of Fas/CD95 to lipid rafts during death receptor-mediated apoptosis in cancer chemotherapy [27], additional reports showed the rapid recruitment of Fas/CD95 to lipid rafts in mouse thymocytes following FasL/CD95L ligation [81], as well as Fas/CD95 clustering in neutrophils [105] suffering its characteristic spontaneous apoptosis [106]. These data suggest that edelfosine-induced death receptor clustering in raft platforms is an exacerbation of a physiological process leading to apoptotic cell death [9].

## CASMER, a novel supramolecular structure favoring protein–protein interactions in Fas/CD95-mediated apoptosis

**Figure 1.**
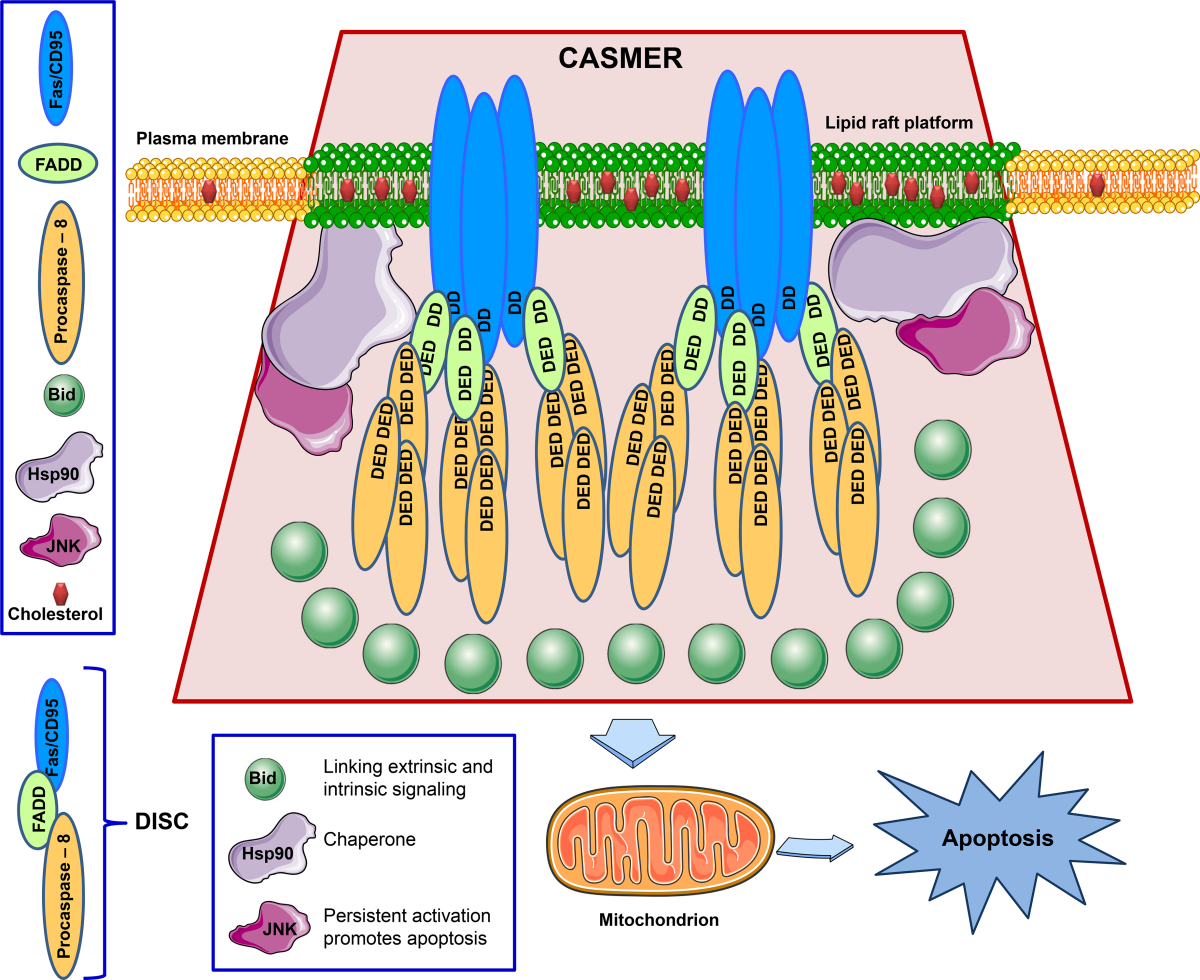
The concept of CASMER. Apoptotic signaling molecules, including Fas/CD95, FADD and procaspase-8, forming the DISC, are brought together in close proximity, through homotypic interactions of DD and DED domains between the DISC constituents, in large cholesterol-enriched lipid raft platforms (highlighted in green) as a result of raft clustering. This leads eventually to a high concentration of procaspase-8 molecules that favors its autoproteolytic activation. Additional downstream signaling molecules, such as Bid, can also be recruited to the CASMER, acting as a bridge between death receptor extrinsic apoptotic signaling and mitochondrial-dependent intrinsic apoptotic signaling, thus potentiating apoptosis. JNK could also be translocated into CASMERs, and persistent JNK activation potentiates apoptosis. The activity of JNK can be protected by chaperones, such as Hsp90, that when redistributed into rafts replace their classical client proteins with other proteins predominant in these proapoptotic rafts. The formation of the supramolecular entity CASMER highly facilitates protein–protein interaction and cross-talk signaling, and eventually favors the generation and amplification of different apoptotic signals, including caspase activation- and mitochondria-mediated processes, that lead to the same outcome, viz., the trigger of apoptosis. Thus, CASMERs act as linchpins from which the apoptotic response is highly amplified. See text for further details.

In the early 2000s, we coined and postulated the concept of CASMER (an acronym of ‘cluster of apoptotic signaling molecule-enriched rafts’) [62–64,102,107–109], in order to represent a raft-based supramolecular entity that act as a hub for the triggering and transmission of apoptotic signaling (Figure 1) [6,9,108–110]. CASMER refers to apoptosis-promoting raft platforms, where death receptors and downstream signaling molecules are brought together in close proximity to facilitate protein–protein interactions and the transmission of apoptotic signals [9]. These CASMERs act as scaffolds for harboring Fas/CD95 aggregates and the subsequent downstream signaling, acting as the linchpin from which a potent death signal is launched [6,9,102,108–110].

It is broadly assumed that apoptosis can be triggered by an alteration of the overall balance between apoptotic and survival signals, viz., the apoptosis/survival ratio. As a consequence of the CASMER concept, it is clear that this apoptosis/survival signaling balance could also be modified by a redistribution and local accumulation of apoptotic molecules in lipid rafts, segregating apoptotic from survival signaling molecules. This concentration of apoptotic molecules, set apart from survival signaling molecules, leads to a dramatic local change in the apoptosis/survival ratio in a specific region that eventually triggers a cell death response. In this regard, it is worth mentioning that the ether lipid edelfosine displaces survival proteins from rafts, such as phosphatidyl inositol 3-kinase/AKT signaling in mantle cell lymphoma cells [49] and Pma1p in yeasts [68,70,111], while it recruits apoptotic molecules in lipid rafts [9], thus segregating apoptotic and survival signaling pathways in distinct membrane domains.

The physiological ligand FasL/CD95L has also been recruited in lipid rafts during the apoptotic response [102,112,113]. In addition to Fas/CD95, additional death receptors, such as TNF-R1 and TRAIL receptors DR4 and DR5, have also been reported to be recruited into lipid rafts in response to several chemotherapeutic agents [9]. Thus CASMERs might contain different death receptors [9]. This co-clustering of death receptors and lipid rafts sensitized cancer cells to their cognate death ligands, including TRAIL [64,114], which is in phase II and phase III clinical trials [2,115].

## CASMERs act as proapoptotic raft scaffolds to harbor signaling molecules facilitating death receptor-mediated apoptosis

Interestingly, some proteins, not necessarily related to cell death processes, could be recruited to apoptosis-promoting rafts, and once there they could support apoptosis in the new proapoptotic microenvironment. Treatment of leukemic T-cell Jurkat cells with edelfosine led to the recruitment of heat shock protein 90 (Hsp90), together with c-Jun N-terminal kinase (JNK) and DISC components, in lipid rafts, but not of the JNK regulators apoptosis signal-regulating kinase 1 (ASK1) and Daxx [116]. Hsp90-JNK clusters in lipid rafts were detected by immunoelectron microscopy, immunoprecipitation, and functional assays, showing a chaperoning role of Hsp90 on JNK-mediated apoptosis when both proteins were translocated into lipid rafts [116]. Interestingly, Hsp90, acting usually as a survival signaling chaperone and being thereby considered a cancer chemotherapeutic target, changed its role in the modulation of cell fate to an opposite outcome, viz., apoptosis, when was recruited to a proapoptotic environment in lipid rafts enriched in JNK and DISC (Figure 1) [116]. This is a stark example of how the function of certain proteins could be affected by the microenvironment in which is located. Thus, Hsp90 may play a role in maintaining the stability of lipid raft-associated proapoptotic signaling upon edelfosine treatment. The putative alteration in the behavior of death/survival regulators, depending on their location in raft platforms, may have important consequences in combination chemotherapy, and should be taken into consideration in order to avoid unwanted effects.

Taken together, a remarkable feature of these CASMER proapoptotic rafts is to harbor signaling molecules bound to yield the same outcome, viz., to trigger apoptosis. This could give point to the following rhyme, which briefly summarizes the main idea of compartmentalization in death receptor-mediated apoptosis:

*proteins conveying a deadly fate,*

*gather together in the same plate*.

**Figure 2.**
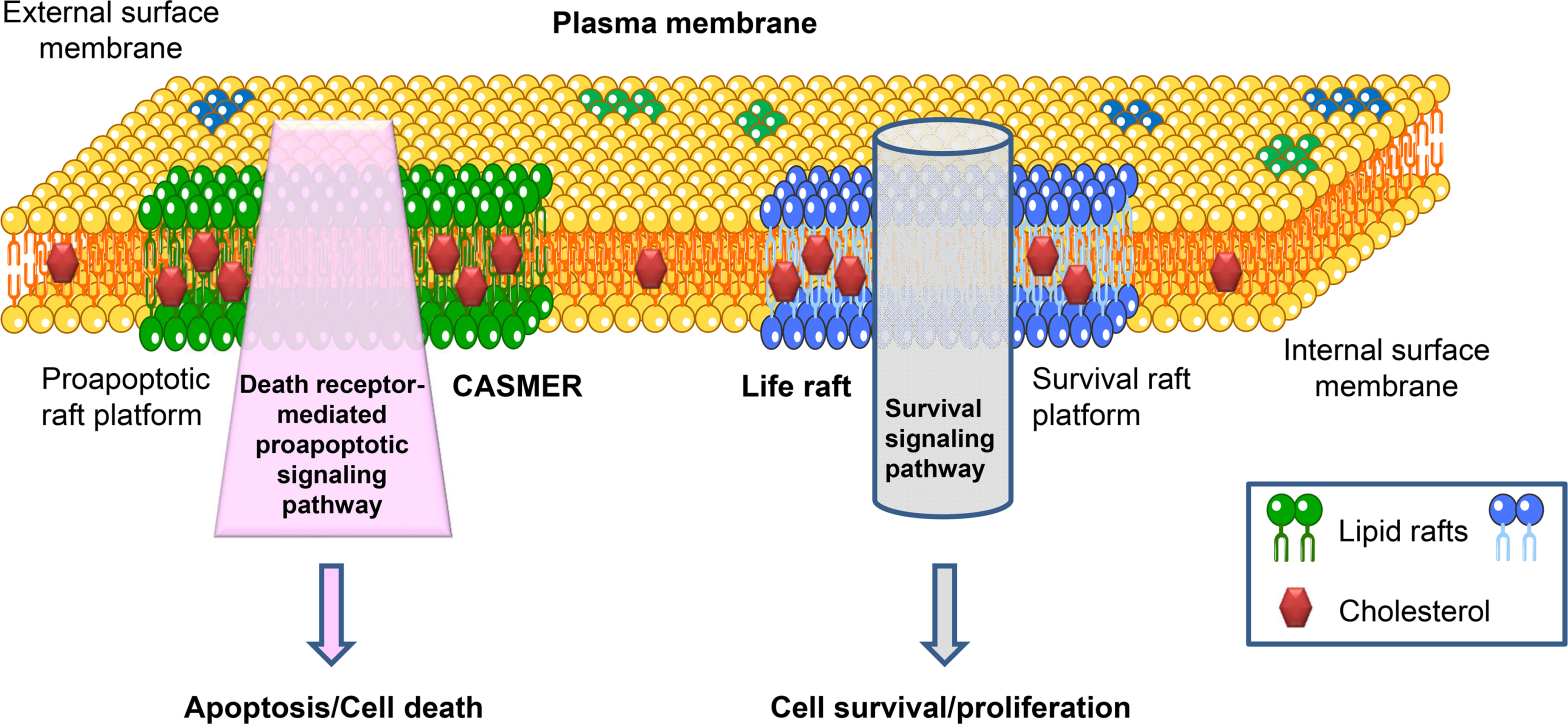
Lipid raft platform heterogeneity in composition and function. Lipid rafts can coalescence to form large cholesterol-rich lipid raft platforms with distinct lipid and protein composition, which harbor specific downstream signaling pathways. Segregation and recruitment of proteins, through mechanisms that are not yet well understood, result in the generation of raft platforms enriched in death-receptor mediated proapoptotic signaling molecules, leading to a proapoptotic raft platform (CASMER), or in survival signaling molecules, leading to a survival raft platform (life raft). See text for further details.

This sums up the whole notion of the CASMER concept (Figure 1). Apoptosis would be triggered by the clustering and close interactions of death receptors and proteins that transmit cell death signals in a limited region of the cell membrane (proapoptotic raft platforms), which segregates cell death from survival signaling molecules. One could envision the cell membrane with different types of raft platforms modulating cell fate, e.g. life rafts and CASMERs, each one enriched in different signaling processes leading to opposite outcomes (Figure 2).

With our CASMER formation theory, it is tempting to suggest that death receptor-mediated apoptosis could be viewed as a kind of a quantum process, rather than a continuum one, in which CASMER would represent the smallest discrete unit related to the generation of an eventual apoptotic response. A certain threshold should be overcome in order to trigger the apoptotic response. The greater number of CASMERs, the strongest apoptotic response is achieved. Thus, the apoptosis ability of a cell type would rely on the amount of CASMERs to be formed as well as on their composition and complexity. The basic protein composition of the functional unit of a CASMER would consist of death receptors, FADD and procaspase-8 to form the DISC in aggregated rafts. Ultrastructural electron microscopy evidence for the formation of aggregates of lipid rafts, containing Fas/CD95 and DISC, has been shown in leukemic Jurkat T cells upon treatment with edelfosine [63,107], thus visualizing a CASMER in a hematological cancer cell. Additional genetic and pharmacological approaches demonstrated the functional role of CASMER, viz., to promote apoptosis through the essential requirement of both cholesterol-rich lipid rafts and the DISC components [62–64]. A further stage in CASMER complexity would entail the recruitment of additional downstream signaling molecules involved in apoptosis, which could enhance the proapoptotic capacity of the raft platforms. In this regard, edelfosine is able to promote the recruitment of JNK and Bid into lipid rafts [62,64], that is into the so-called CASMERs, in several hematological cancer cells. In this regard, the apoptotis ability of a determined cell type could be quantifiable. The higher the number of proapoptotic proteins recruited to CASMERs, the more potent the apoptotic response would be. The proapoptotic Bcl-2 family member Bid, acting as bridge between Fas/CD95 signaling and mitochondria [117], has been found to be translocated to membrane rafts after treatment of distinct leukemic cells with different antitumor drugs [62,64,100,102,107], thus pointing out a putative role of lipid rafts as a linker between death receptor-mediated extrinsic and mitochondria-mediated intrinsic signaling pathways in apoptosis (Figure 1). This facilitates apoptotic signal transmission from the cell membrane to the interior of the cell, amplifying the cell death signal.

The antitumor agent edelfosine, which accumulates in lipid rafts as stated above, has also been located in the endoplasmic reticulum [118–122] and mitochondria [50,123,124] in different cancer cells. This could suggest a raft-mediated link between plasma membrane rafts and internal subcellular organelles during the apoptotic response.

## Nonapoptotic functions of Fas/CD95 and lipid rafts as a regulatory element

Fas/CD95 is not only able to induce apoptosis but also can trigger nonapoptotic signals leading to cell survival proliferation, inflammation, cancer growth and metastasis [25,26,125,126]. The fact that engagement of the same Fas/CD95 receptor, devoid of enzymatic activity, could lead to different and even opposite outcomes suggests the possibility of different ligands and/or fine-tuned control of Fas/CD95 through its aggregation, conformation, ability to interact with different signaling pathways, and post-translational modifications of Fas/CD95 and DED proteins [8,25,126]. The capacity of Fas/CD95 to promote different outputs is a consequence of triggering different sets of protein–protein interactions in a cascade way. In this regard, it is tempting to suggest that the distribution of Fas/CD95 within certain plasma membrane domains, such as lipid rafts, could account for such a broad-range of cell signaling sharing a close microenvironment with different proteins [6]. This would highlight the key role of lipid raft reorganization in cancer therapy [9,61,127]. In this regard, the assembly of TRAIL receptor-DISC in lipid rafts led to apoptosis in non-small cell lung carcinoma (NSCL) cells, whereas TRAIL receptor-DISC assembly in the nonraft phase of the plasma membrane led to the inhibition of caspase-8 cleavage and the activation of NF-κB and extracellular signal-regulated kinase 1/2 (ERK1/2) in TRAIL-resistant NSCL cells [128]. Furthermore, inhibition of the survival signaling phosphatidylinositol 3-kinase (PI3K) signaling pathway sensitized tumor cells to death through the redistribution of Fas/CD95 into large platforms of membrane rafts [129].

## Conclusions

Accumulating evidence shows that lipid rafts serve as scaffolds for the clustering of Fas/CD95 and DISC, leading to the induction of death-receptor mediated apoptosis, as well as to the recruitment of additional downstream signaling molecules related to cell death, thus forming the so-called CASMER. Subsequent signaling molecules accumulate in this novel structure, behaving as an iceberg, in which the larger part of structure lies inside the cell and communicates with other subcellular structures (Figure 1). CASMER represents a novel raft-based supramolecular entity, which seems to play a critical role in the regulation of death receptor-mediated apoptosis, acting as death-promoting scaffolds where death receptors and downstream signaling molecules are brought together, thus facilitating protein–protein interactions and the transmission of apoptotic signals. This is of particular importance in signaling pathways, like the Fas/CD95 route, where the first stages depend exclusively on protein–protein interactions between molecules lacking enzyme activity. Formation of CASMERs can be induced physiologically and pharmacologically, independently of FasL/CD95L, and represents a new way to promote cancer cells to kill themselves using their own cell machinery. The generation of proapoptotic raft platforms highlights the importance of lipid rafts as scaffolds for harboring proteins that depend on short-range interactions to transmit signals. Moreover, signaling molecules might change their regulatory and interacting features when redistributed between a raft platform and a nonraft microenvironment. A major message of these observations is the suggestion that death receptor-mediated apoptosis requires membrane platforms to be furnished with the appropriate molecules to trigger apoptosis. This could be extrapolated to other signaling pathways, leading to the general hypothesis that specific signaling, involving protein–protein interactions and/or protein complex formation, should require specific physical scaffolds/platforms of limited areas, used as meeting points, where all the proteins involved in a particular signaling route could be gathered in order to interact each other and deliver determined signals. In this raft-mediated signaling compartmentalization, many open questions remain to be solved. What are the mechanisms driving proapoptotic raft platform formation, raft clustering, and signaling compartmentalization in raft platforms? How Fas/CD95 and downstream signaling molecules are recruited into proapoptotic raft platforms? Can Fas/CD95-DISC assemblies distribute in different membrane rafts with particular protein compositions or to nonraft domains leading eventually to apoptotic or nonapoptotic signals? How many different types of raft platforms can coexist in a cell type? Could different types of rafts be interchangeable by protein or lipid redistribution? Do different raft platforms influence or transform each other? How signaling molecules are segregated between proapoptotic and survival rafts? What is the role of cholesterol and lipidome in spatiotemporal compartmentalization and raft coalescence, as well as on raft-mediated interplay between plasma membrane and different subcellular organelles? How are these raft-mediated signaling processes turned on and off? Answers to these questions will definitively provide new insights in our understanding of the modulation of cell fate, with consequences in the treatment of human diseases, where cell death regulation plays a critical role, such as cancer and neurodegenerative diseases.

## Perspectives

- The importance of the field: The recruitment of death receptors and apoptotic signaling molecules in lipid rafts provides a way to facilitate their interactions and trigger apoptosis. This process can be modulated physiologically and pharmacologically, opening up a promising avenue in cancer therapy.
- Summary of current thinking: Cholesterol-rich lipid raft platforms constitute scaffolds for the recruitment and activation of the death receptor signaling pathway, providing opportunities to develop novel therapies for cancer and apoptosis-mediated diseases. The ability to generate a raft-mediated apoptotic response will depend on the relative presence of apoptotic molecules to be recruited into the raft platforms. Lipid rafts might act as linkers between the plasma membrane and different intracellular subcellular organelles in order to transmit molecules and signals.
- Future directions: A deeper understanding of how death receptors and downstream signaling molecules are recruited into the raft platform, thus leading to the triggering of apoptosis, will provide opportunities to develop novel therapies in cancer and apoptosis-mediated diseases, as a result of apoptosis modulation.

## Abbreviations

CASMER: cluster of apoptotic signaling molecule-enriched rafts
DD: death domain
DED: death effector domain
DISC: death-inducing signaling complex
DR4: death receptor 4
DR5: death receptor 5
DRM: detergent-resistant membranes
FADD: Fas-associated death domain-containing protein
Hsp90: heat shock protein 90
JNK: c-Jun N-terminal kinase
MβCD: methyl-β-cyclodextrin
NF-κB: nuclear factor kappa-light-chain-enhancer of activated B cells
NSCL: non-small cell lung carcinoma
TNF: tumor necrosis factor
TNFRSF: TNF-receptor superfamily
TRAIL: TNF-related apoptosis-inducing ligand
